# Supplementary material for: Variation in base composition underlies functional and evolutionary divergence in non-LTR retrotransposons
Source: Mob DNA. 2020 Apr 7;11:14. doi: 10.1186/s13100-020-00209-9 (PMC7140322; doi:10.1186/s13100-020-00209-9)
Supplement: Supplementary file 2 — Additional file 2. Phylogenetic relationships among Rex1 elements based on the entire ORF2. [file 13100_2020_209_MOESM2_ESM.pdf]

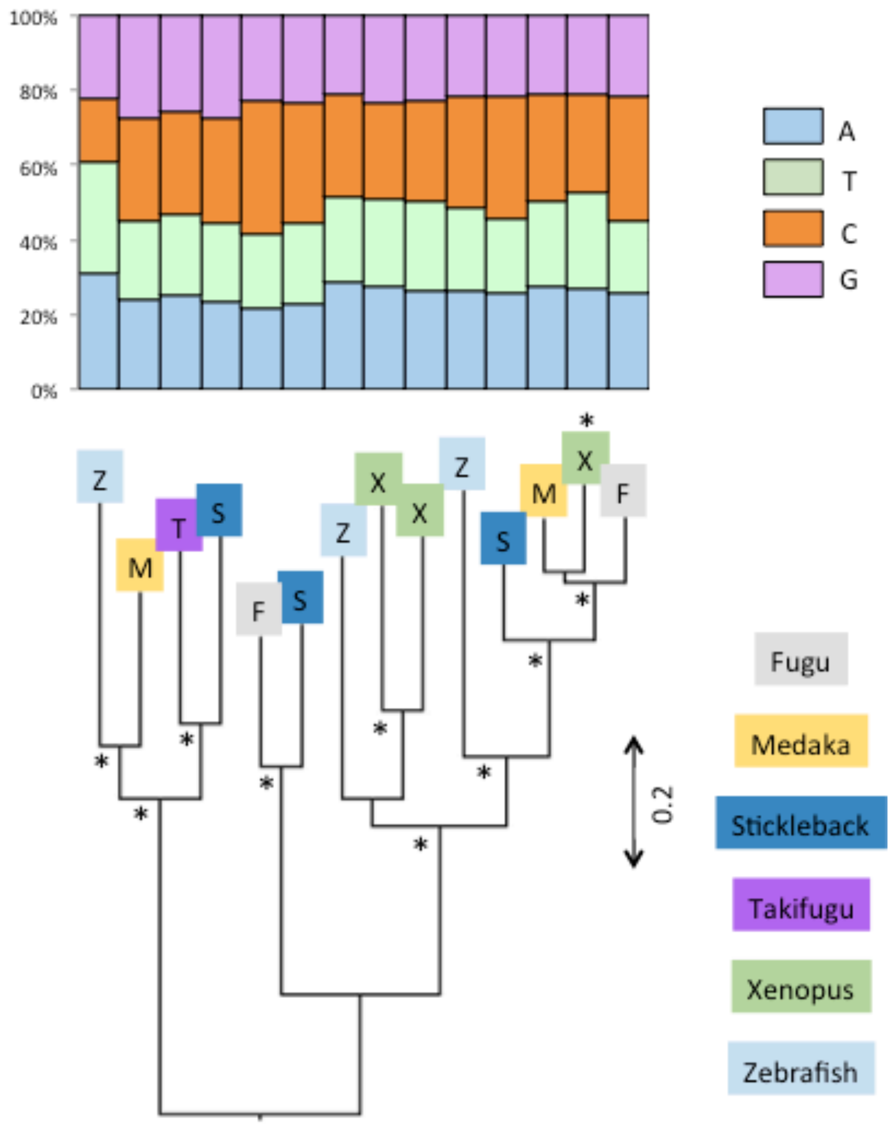

Supplementary material 2 – Phylogenetic relationships among Rex1 elements based on the entire ORF2. The base composition of each element is shown above the tree. The tree was built with the maximum likelihood method using the LG+G+I+F model of mutation and its robustness was assessed by 500 bootstrap replicates. Nodes that are supported by bootstrap values higher than 70% are indicated with an asterisk. The frog element suspected of HGT is indicated with an asterisk.
