# Supplementary figures and images for: Variation in base composition underlies functional and evolutionary divergence in non-LTR retrotransposons
Source: Mob DNA. 2020 Apr 7;11:14. doi: 10.1186/s13100-020-00209-9 (PMC7140322; doi:10.1186/s13100-020-00209-9)

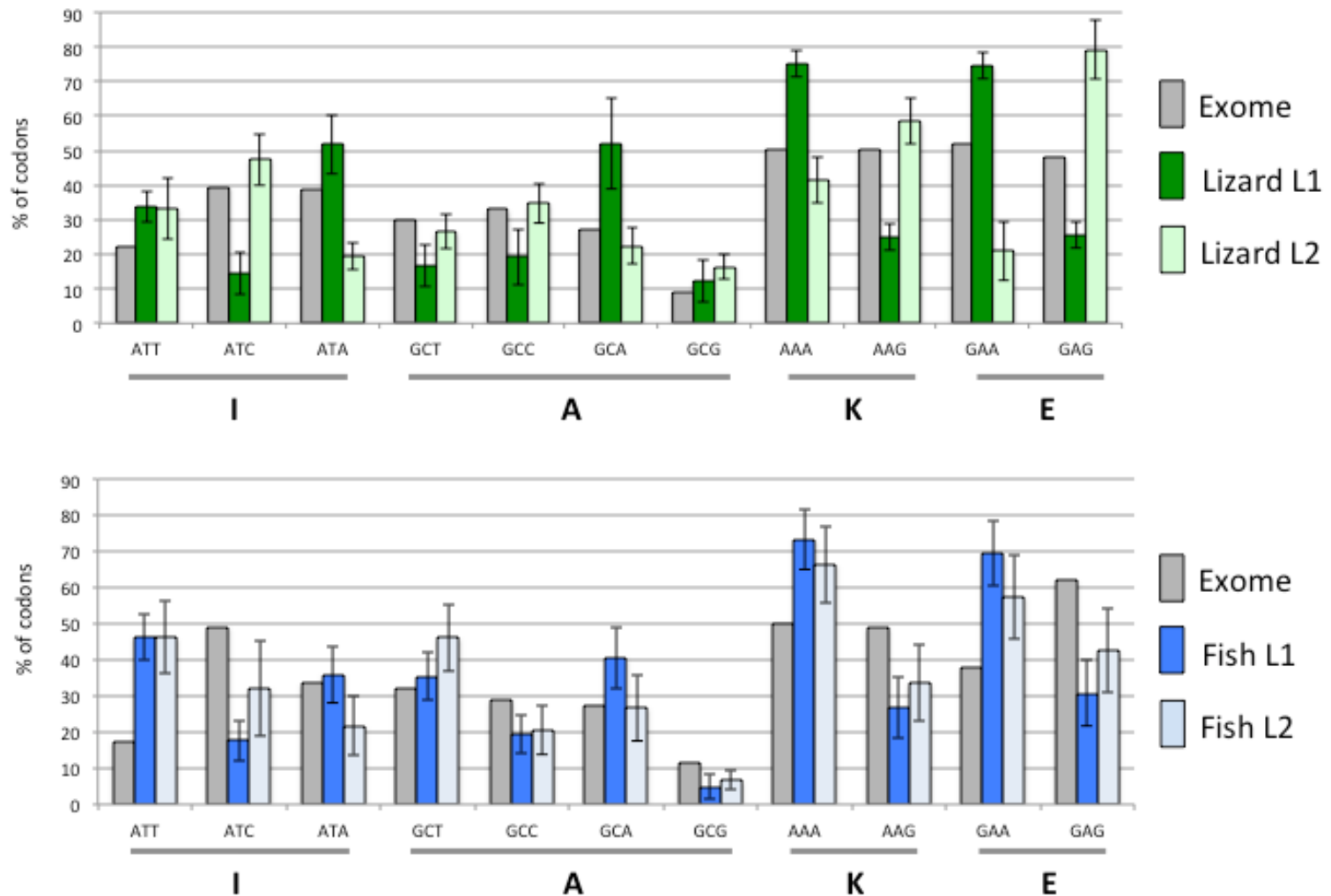

Supplementary material 3 – Codon usage in ORF2 for four amino acids in fish and lizard L1 and L2.

Supplement: Supplementary file 3 — Additional file 3. Codon usage in ORF2 for four amino acids in fish and lizard L1 and L2. [file 13100_2020_209_MOESM3_ESM.pdf]

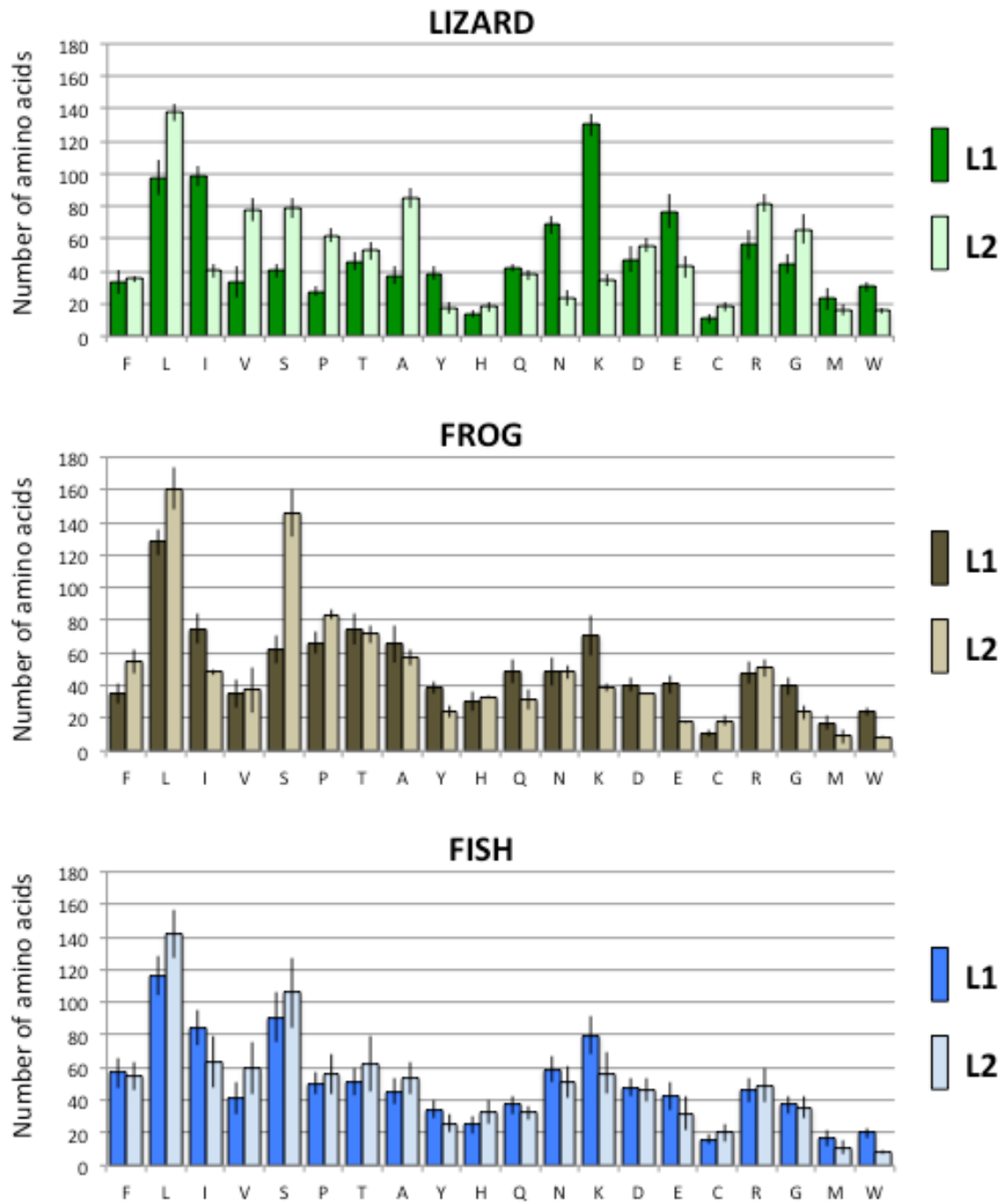

Supplementary material 5 – Number of each amino acid in L1 and L2 ORF2 in lizard, frog and fish.

Supplement: Supplementary file 5 — Additional file 5. Number of each amino acid in L1 and L2 ORF2 in lizard, frog and fish. [file 13100_2020_209_MOESM5_ESM.pdf]

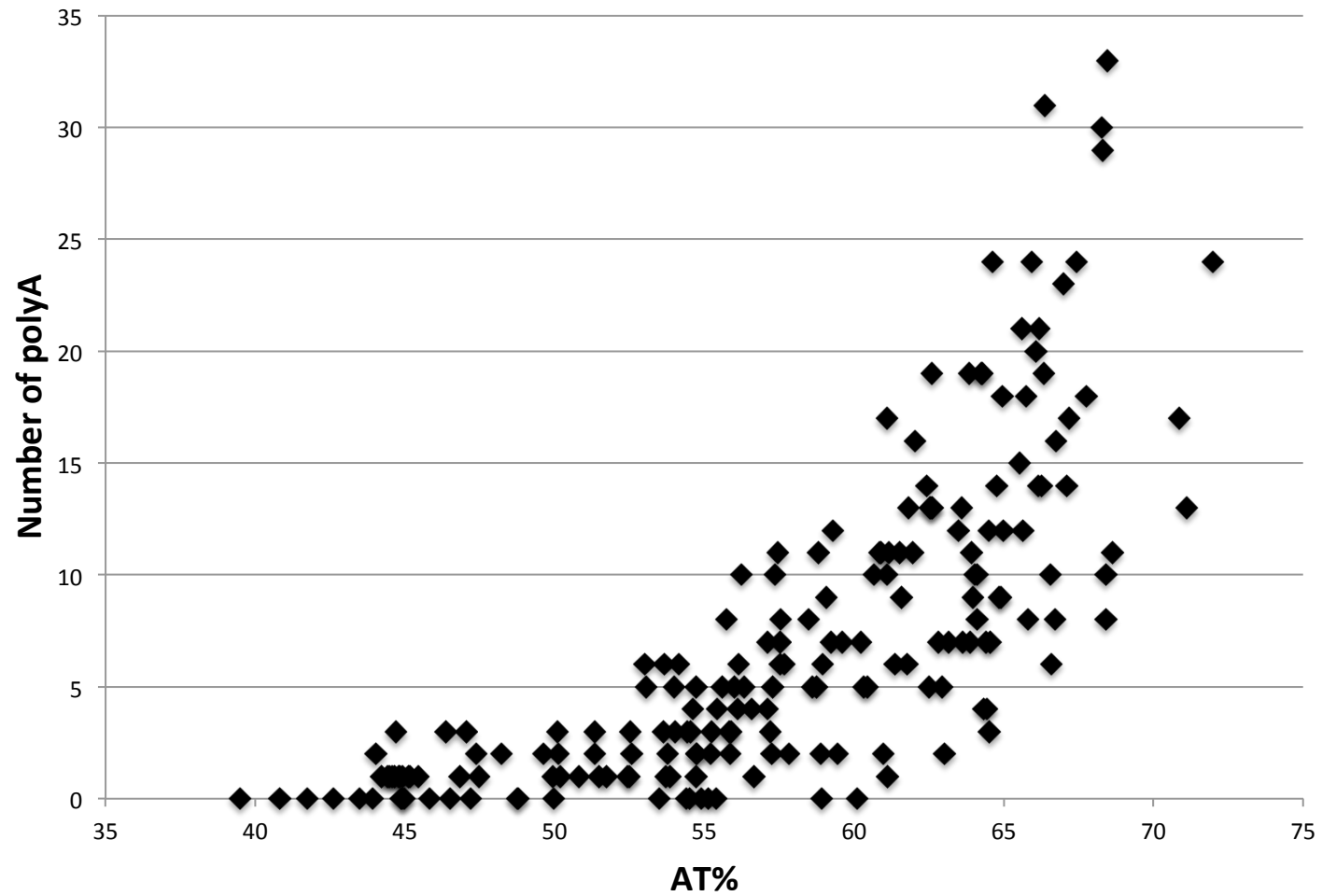

Correlation between the content in AT and the number of predicted poly-adenylation signals

Supplement: Supplementary file 6 — Additional file 6. Correlation between the content in AT and the number of predicted poly-adenylation signals. [file 13100_2020_209_MOESM6_ESM.pdf]
