# Supplementary material for: Variation in base composition underlies functional and evolutionary divergence in non-LTR retrotransposons
Source: Mob DNA. 2020 Apr 7;11:14. doi: 10.1186/s13100-020-00209-9 (PMC7140322; doi:10.1186/s13100-020-00209-9)
Supplement: Supplementary file 4 — Additional file 4. Relationship between base composition at the 3rd position of codon and codon usage statistics. [file 13100_2020_209_MOESM4_ESM.pdf]

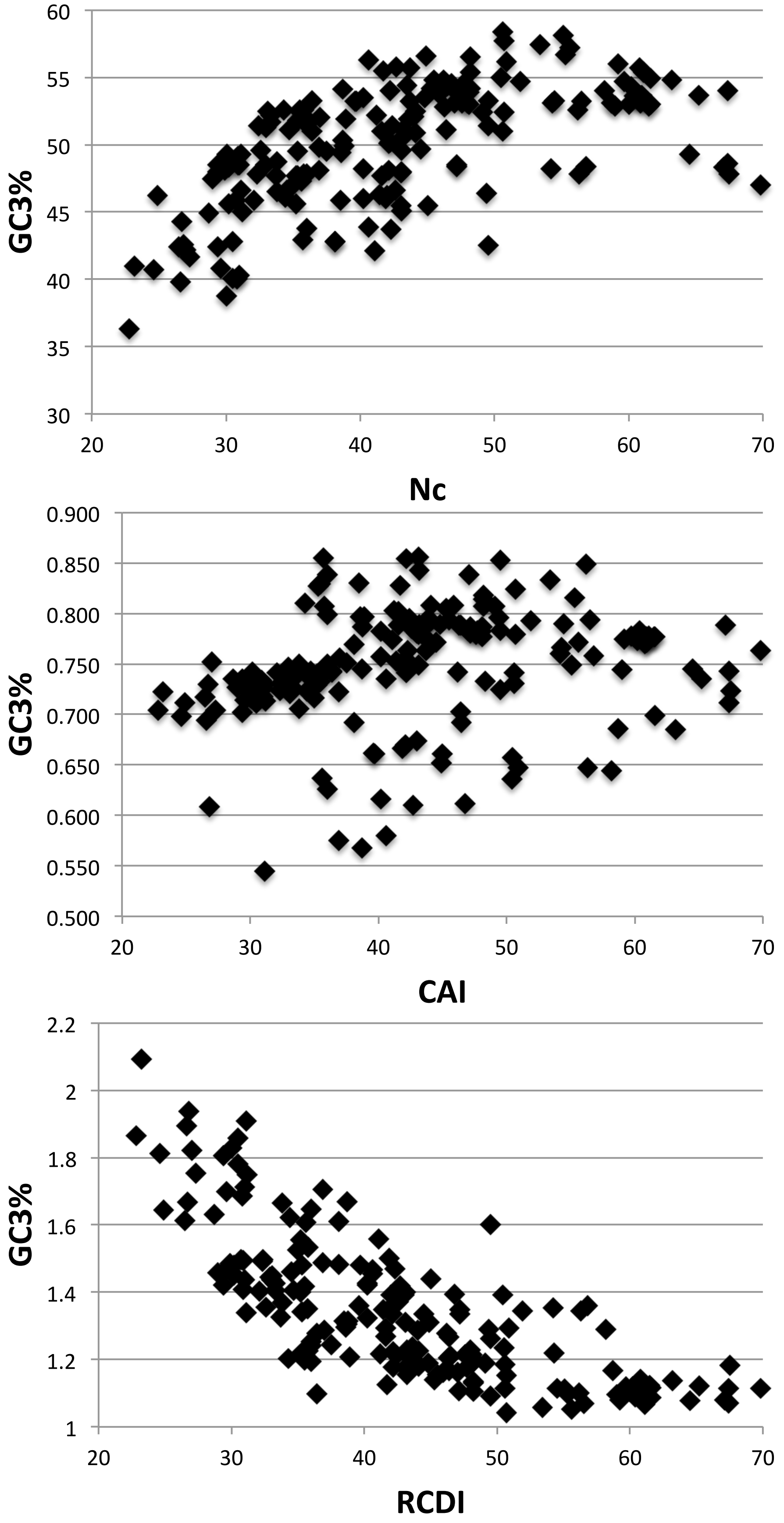

Relationship between base composition at the 3rd position of codon and codon usage statistics.
